# The Prevention of Contagious Pleuro-pneumonia in Cattle

**Published:** 1884-10

**Authors:** 


### ART. XXVI.—THE PREVENTION OF CONTAGIOUS PLEURO-PNEUMONIA IN CATTLE.

*A Contribution to the Question of Inoculation and Stamping Out.*

BY DR. OEMLER.

(Archiv. f. Thierheilkunde, vol. x. Berlin. 1884.)

### INOCULATION.

The principle that inoculation of the elements of this disease upon healthy cattle would prevent natural infection, was first brought into practical importance by Willems in 1852, though it had been suggested by Hausmann in 1819 ; but nevertheless, to Willems belongs the credit of drawing general attention to this procedure. Willems and his immediate followers claim that the inoculation treatment can only prevent the disease in absolutely healthy animals at the time of the inoculation, while others claim for it a favorable influence upon the course of the disease if resorted to in its earliest stages. Others deny any value whatever to inoculation, while others consider it still to be an open question whether inoculation has any protective influence against natural infection.

It is a well known fact that whenever a general eruption of pleuro-pneumonia contagion occurs in a herd of cattle, it must have been present in a latent form in one or more members of the same, and that even though such animals manifested no outward signs of the disease, still they were in a condition to cause infection of those surrounding them. Therefore, in every herd we have a great or less number of animals which have the disease in the latent form, or by which infection has just occurred, according to the degree and severity of the disease ; the number and situation of those first diseased, according to the time which the latter have been members of a given herd ; or the size of the stables, and other influences which play no unimportant role in the extension of this pest among cattle, such as stabled or on pastures, ventilation, temperature, manner of feeding, etc.

The observer would fall into a most serious error should he assume that all apparently healthy animals of a herd in which pleuro-pneumonia was known to exist, were still free from that disease. I have frequently found as high as 60 per cent. of such animals—which, *intra-vitam*, displayed no other ascertainable indications of pulmonary complications than a slight and occasional cough—to have the distinct microscopical evidences of pleuro-pneumonia.

In some such cases I have found not only centres of recent formation, but old ones in the lungs; but also such which were entirely sequestered and ante-dated by the day or even year of their introduction into the herd in question, and which demonstrated no sign of being ill, and hence no one ever entertained a suspicion of their being the means by which the disease was introduced into the herd.

"An ox stood between twelve cows in a stable; on slaughtering, pleuro-pneumonia was found, though no one suspected it before. Soon after all the cows were killed, and in all of them the disease was apparent in its early stages. Numerous other illustrations of this nature could be quoted."

If inoculation had been resorted to in such a case as the above, i. e., immediately after the ox had been slaughtered and the danger to the cows revealed, it will be evident the forthcoming disease could not be laid to the preventive procedure, nor could any immunity to natural infection upon known exposure be credited to the same in the future. It is not claimed by Willems and many of his followers, for the inoculation, that it can exert any favorable influence upon already existing disease, even though it be present in the very earliest stages; but that where no disease is present, it can protect the bovine organism from natural infection.

Others, on the contrary, claim that preventive (?) inoculation *does* exert a favorable influence upon the cause of the disease, if the procedure is resorted to at a timely moment; they even go so far as to assert that the disease is checked in its progress, and the retrogressive processes at once occur. All proof is wanting for such assertions. Their authors appear to have ascribed some derivative influence upon the processes in the lungs, the inflammation occurring at the locus inoculationis.

It would be far more logical to assume that, if the lymph in reality contains the active germs of pleuro-pneumonia, in such cases the course of the disease would be accelerated. The Holland Commission, as well as some other observers, seem to incline to support this idea, having observed that the majority of those animals perished in which pleuro-pneumonia was present at the time of the inoculative treatment. It has also been reported that the symptoms of pulmonary complications frequently followed inoculation. Pasteur's experiments have shown that inoculation hastened the results upon animals already infected with anthrax, so that he emphatically cautions against the exposure of animals destined for preventive inoculation, to any unfavorable influences.

Against the assumption that an exacerbation of the processes of an already existing pleuro-pneumonia occurs through inoculation, is the fact that no one has yet seen the actual disease follow that treatment in known healthy animals; hence it is very doubtful if the virus virtually contains the same elements which cause the disease under natural circumstances. The contrary is the case in Pasteur's treatment of anthrax, for his vaccine frequently produces that disease.

It matters not what side we take upon this question, all must unite in looking upon inoculation as having an unfavorable rather than the contrary influence upon already existing pleuro-pneumonia in the bovine organism.

If, however, the pest assumes a favorable course subsequent to the inoculation, it is not to be accredited to that procedure, but to favorable influences of another character. In such cases the question to decide is, *if the inoculation of absolutely non-diseased cattle is capable of promoting natural infection?* According to my notion, this question cannot be answered in a positive manner.

The advocates of inoculation generally assume that—as with vaccination, so with inoculation against pleuro-pneumonia—prevention of natural infection can only then occur when distinct inflammatory swelling follows the operation; the same must be repeated when this does not result. Experience has shown, however, that as a rule these re-active tumefactions first appear in the course of eight days to four weeks, and occasionally even later. Accordingly, all animals by which this result fails must still be open to natural infection. Others, on the contrary, do not consider this inflammatory swelling at the point of inoculation of any value, but rather look upon a general absorption of the introduced virus as the manner in which the prevention results.

Even though we admit this assumption, still it must be admitted that the inoculative treatment does not act instantaneously, but that some time must elapse for the generalization or organization, if the tissue is allowable, of the virus before immunity against natural infection can be obtained. During this period the animal would still be open to infection from natural causes.

As previously mentioned, our experience has shown that old and newly-developed processes may be present in the lungs of the same animal at the same time; or in other words, infection or freshly-developed conditions may be present in the lungs even after inoculation has been resorted to, or before its external symptoms have disappeared.

In anthrax, immunity is not claimed to follow preventive inoculation until the symptoms of that procedure have entirely disappeared.

There can, therefore, be no doubt that in pleuro-pneumonia immunity cannot be looked for unless inflammatory indications occur in all inoculated animals. Hence, we cannot ascribe the cessation of the disease to inoculation when it follows soon after inoculation.

As is well known, pleuro-pneumonia often ceases to give any visible indications of illness when it comes to a conclusion in its chronic form—that is, it runs its course without any marked phenomena, notwithstanding pathological disturbances in the lungs of no inconsiderable degree. This is proved by microscopical observations made upon herds in which pleuro-pneumonia has been suspected.

"In a stable of oxen, one of a newly purchased lot was killed on account of pleuro-pneumonia, the lungs being found in a condition of old and fresh invasion characteristic of this disease. The other remained apparently healthy; but as they were slaughtered in the course of the following winter, consolidation was found in the lungs of all of them. This has been frequently found to be the case in other herds at various places, of which the records of the abattoirs at Berlin, Köln, and other places, give sufficient evidence. In 1881 the authorities of Holland commanded 189 cattle to be slaughtered, all of which were apparently healthy. Of them, 87 head (46 per cent.) had the positive pulmonary indications of pleuro-pneumonia. Of 664

from the same locality in which the disease was suspected, 153 were found infected."

I have confirmed myself that the disease frequently assumes this occult non-suspected form in many cases where all the cattle present were killed in order to stamp out the disease. The disease frequently assumes this mild course, especially among cattle when first attacked, and we find the pest assuming a retrogressive course in many animals when our attention is first called to it. We often find the disease assumes such a course under favorable external conditions, and that but a very insignificant loss follows its invasion in such herds.

It frequently follows that the disease does not manifest itself in any marked degree in a large number of animals in a given herd, though no inoculative treatment is resorted to. This has caused some to assume that cattle were susceptible to a non-contagious form of the disease.

"In a certain herd of 30 head, two members acquired the disease from an animal that was considered to have recovered from it. The suspected animals were at once isolated, and the stable disinfected. Other cases did not occur."

"In a stable of twenty-four cows, one bull, and three calves, four cows became diseased, of which three were slaughtered and one recovered. All the others remained well. Roloff has collected a list of observations, in which only 5 per cent. of the animals exposed to infection acquired the disease."

"On a farm having 80 head of cattle, only eight had the disease, of which two died, two were slaughtered, and four recovered."

Veterinary literature is full of such reports, showing that, like glanders, the susceptivity to the contagion of pleuro-pneumonia is not a general property of all the individuals of the bovine race. I have frequently made similar observations among cattle belonging to large herds, which had never been inoculated, and among which no one had suspected the presence of pleuro-pneumonia until they had perished or died from other causes—anthrax—when the presence of squeeters, old pleuro-pneumonia, and pathological processes were revealed. The owners were often astonished at such revelations, but have asserted that on some occasions previously they had noticed want of appetite and coughing among many members of their herds ; also that many otherwise apparently healthy animals had been troubled with a cough, which had been attributed to unfavorable climatic conditions. Others have reported similar observations, among them Weinmann, who describes under the head of undoubted pleuro-pneumonia which no one had suspected, that of a herd of twelve which were sold as healthy, he found a cow with every indication of a sequestered pleuro-pneumonia centre in the lungs.

Such cases go to make one skeptical of the protective value of inoculation. They have been too frequently ignored by the advocates of this procedure. Hildebrand, a very earnest advocate of inoculation, asserts that never more than 10 per cent. of the animals exposed to infection remain unnoticed, or pass so through the disease. Such a proportion of infection does not at all correspond to the general experience, and goes to show that Hildebrand has only noted cases that had a malignant course, and that he at once undertook a general inoculation of herds, and has but seldom made an accurate study of the natural course of the disease. Other defenders of the inoculative procedure have not passed over these mild cases, but place an especial value upon inoculation, because of a favorable course in herds, where the disease has long been stationary, and many animals have acquired immunity from having had this disease in a mild form, rather than having it produced by inoculation.

Other objections can, however, be raised against the value of inoculation as a prevention, or against its having a favorable influence upon the course of the natural disease :

1. As is well known, we find pulmonary diseases in cattle which bear a strong resemblance to contagious pleuro-pneumonia, both in their clinical and microscopical phenomena, and which, as the records of our literature show, have been frequently mistaken for the specific disease. I have personally met with cases in which veterinarians have declared such cases to be pleuro-pneumonia contagion ; also such in which they have subjected the remainder of the herd to the inoculative treatment. One of these cases was in the practice of a veterinarian whose reputation as an expert in pleuro-pneumonia extended far beyond the limits of the district in which he generally practised, which shows that such differentiation is not always the easiest thing in the world.

If in such cases inoculation be resorted to, and no more cases follow, the natural conclusion would be to credit this procedure with preventive forces which did not belong to it. It is therefore a very questionable procedure to claim all such cases for inoculation, as has been done by the advocates of that measure.

2. In the early days of my practice, when cattle diseased with pleuro-pneumonia were only exceptionally slaughtered, and appropriate protective regulations but seldom executed, I have known many cases of pleuro-pneumonia in which inoculation was resorted to immediately upon the slaughtering of the first suspected cow in a herd, the disease coming to an end very soon after. In such cases, many cattle were either killed or sold to butchers on account of suspicious phenomena, such as a cough, emaciation, etc. In the course of time it would seem to have passed from memory that pleuro-pneumonia had been present; yet in entirely unsuspected animals the disease would again break out. This would occur notwithstanding inoculation had been resorted to, and the animals carried the marks of its successful accomplishment on their tails.

I have even more frequently observed in stables where for a longer or shorter period, sometimes for years, the disease has been apparently stamped out, inoculation, through cleansing and disinfection, having been resorted to in unison with slaughtering of many animals, still it would again break out, although the newly-acquired animals were derived from places known to be free from the pest. It has also been observed in Holland that the disease would again break out in forms where the most exact inoculation and vigorous stamping-out had been resorted to.

Such testimony sufficiently demonstrates the impossibility of inoculation thoroughly rooting out the disease.

3. It is a well known fact that in so-called pleuro-pneumonia districts, where the owners of cattle have become thoroughly acquainted with the characteristics of this disease, that immediately on its appearance such owners resort to every endeavor to dispose of such cattle, in order to avoid the disturbance caused by the Veterinary Police regulations. By doing this, however, the disease generally comes to the knowledge of the authorities through the market Inspectors. It thus frequently happens that only such animals remain in such herds as have been through the disease in its mild form. If, as is often the case, inoculation is then inaugurated, it naturally follows that many favorable results are recorded by its advocates.

Such experiences have undoubtedly served to largely increase the confidence of inexperienced and unreflecting breeders, as well as enthusiastic friends of inoculation, in the value of the treatment.

I was at first a confirmed believer in the value of inoculation as a preventive measure against pleuro-pneumonia, but the more experience I gained, the more skeptical have I become with regard to it—a view which is also held by many other members of the profession, Roloff, Kloos, Trasbat, *et al.*

The malignancy which frequently accompanies the invasion of pleuro-pneumonia in certain cases, is not to be attributed to any neglect of inoculation, but rather to constitutional and other conditions. It is well known that no other animal pest offers so many variations in every department as this pulmonary pest of cattle. It may be that local influences of which we have no knowledge, exert a favorable influence upon this disease.

I have frequently observed that the disease assumed a most mild type in certain localities at one time, while at another it has a most malignant course. Others have recorded similar observations. Such variances occurring with and without inoculation, in various parts of Europe, have helped largely to increase the growing skepticism against the preventive importance of inoculation.

Bouley, one of the strongest advocates of inoculation, has recently admitted that it is not always followed by satisfactory results in large numbers of cases which come to practical observation.

According to the friends of inoculation, the unfavorable course which the disease often assumes, even when inoculation has been resorted to, is to be attributed to unfavorable influences exerted by the weather, feeding, etc., which interfere with the absorption of the artificially applied inficiens, or that such material had become polluted with foreign elements, or its character changed, or that the reactive tumefaction had not been treated *lege artis.* To which it can be assumed that, in general, those persons that have executed the inoculations were competent veterinarians, and well versed in the *modus operandi* of this procedure. From my own experience in the matter, I can safely say that my inoculations were executed after the most exact methods of the veterinarian in all the cases where I have had experience, which have led me to doubt the value of the procedure, and that every endeavor was taken to prevent the action of interfering influences. It has never yet been proven that the manner of feeding exerted any influence upon the course of the inoculation. The same is true of the assumed antagonistic action of other micro-organisms which have gained access to the lymph ; nor have we as yet been able to isolate and cultivate any bacteria specific to pleuro-pneumonia. The other assumption, that inoculation has been too lately resorted to, cannot be so easily met, because in many cases the occult stage of the disease is longer than is generally assumed ; and further, because when the disease is diagnosed in a given herd, it is generally the case that the greater number of its members have already become infected, which confirms our already quoted conclusion, *that inoculation has no favorable influence upon the progress of the disease when already initiated in an organism.* This indicates that the inoculative treatment, even though it may produce immunity in non-infected animals, has no ameliorative value in cases where the disease already exists.

The friends of inoculation have further asserted that the disease much less frequently assumes a malignant course when inoculation of the whole herd has been systematically carried out, and conclude, therefore, that the operation has value in this direction. This has not yet been proven by statistical comparisons.

I have frequently observed this pest to break out in herds where for several years "necessary inoculation" had been resorted to in all of the cattle. In all such cases the percentage of diseased animals was about the same in herds that acquired the disease where no inoculation had taken place. This can alone be proven to be correct by the autopsical examination of numerous herds that have been peremptorily slaughtered.

(In the original all these assertions are supported by an immense number of cases that have been gathered through years of practical observation. We have felt obliged to omit them, on account of the length of the article.—TRANSLATOR.)

Another thing which the Inoculationists seem to have entirely lost sight of in their reports of success, is the well known fact that the pest is in most cases introduced into a herd by animals that have it, and that when recognized and a rigid quarantine ordained, it very seldom extends to the cattle in the vicinity, if there has been no antecedent contact; but in general the contrary is the case. On account of the latent character of the disease, its presence is not suspected, and such animals frequently have free intercourse with others for a long time. Pleuro-pneumonia is but seldom extended by means of vehicles on which the inficiens is housed, and so with proper police regulations it is rare that it breaks out in other places in a locality if its recognition has taken place before there has been any interchange of animals, or contact with those of the infected herd.

These facts sufficiently prove that the disease can be *rooted out*, and its extension stopped, without inoculation; hence to properly judge of the value of inoculation, it must be universal, and the utmost freedom allowed in animal traffic. It is an undisputed fact that even in infected districts, by far the greater number of herds have never been inoculated, and still they remain as free from the disease as those of other regions where inoculation is the rule.

It has sometimes been asked is inoculation capable of producing conditions in healthy animals by which pleuro-pneumonia can be generated. Although not referring to this point, Oemler gives some evidence in another direction, which should settle this point forever, viz: *it is impossible.* He speaks of having inoculated the cattle belonging to a large beet sugar factory for a number of years, the average number of head being 650. The inoculation was naturally resorted to as a protective measure. On account of the difficulty of getting lymph in suitable quantities, it often happened that many animals went months before being inoculated, and yet they were in the midst of others that may have been so treated immediately before their arrival. During the years he had the place under observation, the disease never appeared. This shows two things:

1. Inoculation does not produce an infectious condition in inoculated animals.

2. That pleuro-pneumonia did not appear in the herd during this period, was not due to inoculation, but rather that all the newly-purchased animals were healthy, and no accident in introduction occurred.

I (translator) have presented here the essentials of Oemler's article, which is sufficient to attain the end desired, which was to show:

1st. That inoculation is not to be recommended.

2d. That it has not yet been proven that it can produce absolute immunity against natural infection.

That the only way to get rid of the evil in this country is to rigidly stamp it out, kill all the diseased and exposed cattle, burn every stable or shed in which such animals have been kept, and prohibit the building of new *cattle* stables on the same spot for at least one year, and then to take such measures as will prevent the pest ever returning to this country.

